# Long‐Term Trends of Striped Bass Habitat Suitability Under a Temperature–Oxygen Squeeze in Lake Texoma, Oklahoma and Texas

**DOI:** 10.1002/ece3.73736

**Published:** 2026-05-27

**Authors:** Ally M. Whiteis, Shadi Fathollahifard, Isabela Suaza‐Sierra, Hernan A. Moreno, Nishan Bhattarai, Thomas M. Neeson

**Affiliations:** ^1^ Department of Geography and Environmental Sustainability University of Oklahoma Norman Oklahoma USA; ^2^ Department of Earth, Environmental and Resource Sciences The University of Texas at El Paso El Paso Texas USA

**Keywords:** fish habitat suitability modeling, Lake Texoma, striped bass, temperature–oxygen squeeze

## Abstract

Warmer epilimnetic water temperatures and reduced dissolved oxygen levels in deeper water are common stressors for fishes in freshwater reservoirs throughout the summer. We investigated the effects of a temperature–oxygen squeeze—a phenomenon that squeezes fish into a narrow, livable zone—on striped bass from 1995 to 2020 in Lake Texoma. This reservoir is located on the border of Texas and Oklahoma, possesses one of the largest self‐sustaining populations of inland freshwater striped bass in the US, and is of high economic importance to the region. We used a trait‐based approach to quantify (1) how the total volume of suitable habitat for striped bass has changed seasonally and across years, (2) the relative influence of temperature and oxygen on habitat suitability, and (3) whether a temperature–oxygen squeeze has affected the striped bass population and body condition. We found that the volume of suitable habitat for striped bass decreased over the course of each summer and was greatly influenced by day‐to‐day persistence and water level, but did not exhibit a significant linear decline over the years from 1995 to 2020. Additionally, the relative influence of temperature and oxygen on habitat suitability varied depending on the thresholds used to define the temperature–oxygen squeeze. We found no significant linear relationships between habitat volume, striped bass population, and body condition. One hypothesis that would explain this null result is that striped bass in Lake Texoma may be adapted to the seasonally stratified conditions, especially since habitat volume did not show a monotonic decline over time. We also detail other biophysical and statistical hypotheses that might explain why we did not detect an effect of a temperature–oxygen squeeze on striped bass. This study provides insights into future management of Lake Texoma and other reservoir fisheries as global change alters patterns in temperature, oxygen, and reservoir habitat.

## Graphical abstract

This study examined the impact of summer temperature–oxygen squeezes on striped bass habitat, population size, and body condition in Lake Texoma from 1995 to 2020. Although suitable habitat volume consistently declined each summer, with temperature and oxygen influencing habitat in threshold‐dependent ways, we found no clear effects on striped bass population or condition, possibly due to adaptation or other biophysical factors. These findings highlight the importance of incorporating changing thermal and oxygen dynamics into reservoir management and broader biodiversity conservation as climate change continues to alter habitat patterns.

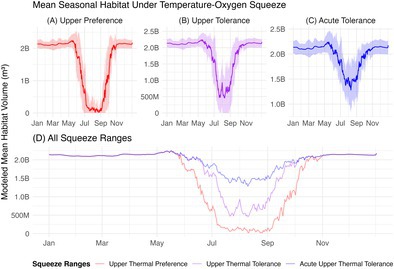

## 1 Introduction

Warming water temperatures and hypoxia are common stressors in lakes and reservoirs around the world (Woodward et al. 2010; Woolway et al. 2022). One mechanism by which these stressors impact reservoir fisheries is through a temperature–oxygen squeeze (Coutant 2013). During summer stratification in eutrophic lakes and reservoirs, surface waters are often too hot for freshwater species, but deeper, cooler waters are hypoxic, resulting in a narrow volume of water that meets species' biological requirements. Thus, the fish are squeezed into a narrow layer between intolerably warm surface waters and intolerably hypoxic deeper waters. Globally, the impacts of this phenomenon have been investigated for many species, including trout, zebra mussels, salmon, sturgeon, and striped bass, among others, with reported changes in water depth distribution, growth and survival, and community dynamics (Berge 2009; Gantz et al. 2022; Kerker 2020; Rowe and Chisnall 1995; Secor and Niklitschek 2001; Young 2016). Specifically, in the southeast and south‐central United States, lab and field studies on striped bass ( *Morone saxatilis* ) have generally supported the hypothesis that large adults will occupy cooler waters below 25°C, if available, and avoid dissolved oxygen (DO) levels of 2–3 mg/L or less (Coutant 1985, 2013). However, this thermal threshold of 25°C has been recognized in some systems as an upper preference limit instead of a tolerance limit, as adult striped bass can withstand higher temperatures (Coutant 2013; Matthews et al. 1985, 1989; Zale et al. 1990).

Coutant led early laboratory and telemetry studies that demonstrated size‐specific thermal preferences of striped bass, with larger adults selecting 16°C–22°C waters and smaller individuals tolerating 20°C–27°C, while avoiding hypoxic conditions (Coutant 1978, 1980; Schaich and Coutant 1980; Waddle 1980). In Tennessee reservoirs, severe squeeze conditions have resulted in crowding into limited cool‐water areas, malnutrition, disease, and die‐offs, but responses differ among study systems (Coutant 1978, 1985). In reservoirs with access to cooler tributaries, tailwaters, springs, or river channels, striped bass occupy true thermal refuges (Coutant 2013). When true thermal refuges are unavailable, striped bass can inhabit mid‐depth areas in reservoirs, often without observed mortalities (Coutant 2013). Nonetheless, feeding reductions and potential condition impacts occur when oxygenated temperatures exceed about 27°C, and prolonged exposure to warm, low‐oxygen conditions can be lethal when food is limited (Zale et al. 1990; Coutant 2013). Thus, site‐specific thermal limits and impacts of a habitat squeeze are determined by the size of adult striped bass, availability of true refuge areas, duration of exposure to warm temperatures, and bioenergetic balance.

Changes in temperature and oxygen in lakes and reservoirs have the potential to reduce the suitable habitat volume for striped bass, especially within the humid and subtropical areas of the US. One such reservoir is Lake Texoma, located on the border of Oklahoma and Texas. Recreational fishing supports Oklahoma and Texas economies by generating approximately $3.04 billion and $7.7 billion annually, respectively (American Sportfishing Association and Southwick Associates 2023; York 2024). Lake Texoma, known as the “Striper Capital of the World,” is one of the few reservoirs in the United States with a naturally producing striped bass fishery, attracting millions of people to the lake every year (Mauck 1986; Sager et al. 2024). Over one million striped bass were introduced to Lake Texoma from South Carolina, Virginia, and New York populations between 1965 and 1974, and natural reproduction was verified in 1975 (Hysmith et al. 2008; Sager et al. 2024). Striped bass also play an essential role as a top predator, controlling prey populations, and are an indicator of ecosystem health (Fay et al. 1983). Specifically, gizzard and threadfin shad represent most of the striped bass' diet, and winter kills of these forage species have negatively impacted year‐class strength (Sager et al. 2024). Thus, Lake Texoma's striped bass fishery is economically, recreationally, and ecologically important to the region and beyond. Lake Texoma also serves as a source of water for municipal, industrial, and environmental services by generating hydropower, increasing economic growth, supporting freshwater ecosystems, and encouraging recreational opportunities.

Fisheries managers suggest that striped bass are at risk of overfishing, habitat loss, changes in prey abundance, disease, and the negative consequences of global warming (Sager et al. 2024). While most fish species in the south‐central U.S. are drought‐tolerant and have broad thermal tolerances, the combination of climatic droughts and accelerating human water use can create conditions for a mega‐drought (Fausch and Bramblett 1991; Godfree et al. 2019; Miranda et al. 2020). For example, low inflows into Lake Texoma caused a severe drought from 2011 to 2014, resulting in a decline in fish catch from 2014 to 2016 (Sager et al. 2024). Additionally, Lake Texoma is situated in the Red River Basin, where severe drought is expected to worsen in the west, and heavy rainfall events are predicted to increase in the east under both high and low emissions scenarios (Bertrand and McPherson 2018). These future changes in climate and hydrology will result in a patchy spatial distribution of water stress across the basin (Sabzi et al. 2019). In combination with ecosystem modifications, water scarcity can result in declining freshwater biodiversity and changes to fish assemblages (Miranda et al. 2020; Vörösmarty et al. 2010). This could greatly impact Lake Texoma and the broader Red River Basin region, as it relies on the many benefits that fish species like striped bass provide.

To cope with these climatic changes, striped bass may migrate within reservoirs, though limited suitable habitats due to a temperature–oxygen squeeze can restrict their movement (Coutant 2013; Daufresne and Boet 2007). Striped bass migrate upstream in the Washita and Red River arms during the spring to spawn (Summers 1982). While some medium and many small striped bass remain in warmer up‐lake areas, large adults move down lake by late May, where they reside deep in the main basin throughout the summer (Matthews et al. 1989; Sager et al. 2024). Large adult striped bass located by transmitters, echolocation, and gillnets occupied mid‐depths near the dam, and their movements within the water column became more constricted with higher surface temperatures and more hypoxic conditions in late summer and early fall (Matthews et al. 1985, 1989; Summers 1982). As surface temperatures ranged from 28°C to 30°C, they occupied the coolest areas directly above the chemocline where temperatures were close to 28°C. Despite exposure to high temperatures, no apparent mortalities were observed for adult striped bass in Lake Texoma during these studies (Matthews et al. 1985).

Large striped bass in Lake Texoma reside in the main basin during the summer due to the lack of available refuge sites, which are discrete, cool locations with ample oxygen (Coutant 2013; Matthews et al. 1989; Summers 1982). While striped bass in systems with true refuges will migrate to tributaries in the summer, those in Texoma remain in the lower lake as it stratifies at around 11–14 m compared to around 9–12 m in the Washita River Arm (Coutant 2013; Sager et al. 2024). This allows for a greater percentage of cool, oxygenated waters. Furthermore, intentional releases from a floodgate adjacent to the location of hydropower generation along the dam in Texoma facilitate oxygen uptake, influencing tailrace water quality and providing a localized refuge for striped bass during lake stratification, reducing stress and subsequent mortalities (Ashby et al. 1999; Sager et al. 2024). However, once in the tailrace, there is no fish lift for striped bass to return to the reservoir. These studies of fish habitat usage, along with other fish biotelemetry studies of reservoirs in the south‐central US, have been conducted to better understand the spatial distribution of striped bass under a temperature–oxygen squeeze (Coutant 2013). Despite the depth of research, there has been a lack of habitat modeling utilizing this hypothesis, which is a necessary step to assess the vulnerability of reservoir fish habitats under climate uncertainty (Coutant 2013; Miranda et al. 2020; Olden et al. 2010). Reservoir water quality models and habitat suitability models can be used to identify future limiting habitats, allowing for fisheries and reservoir managers to determine mitigation strategies that conserve striped bass populations and fish biodiversity.

This research aims to develop a habitat suitability model for managing reservoirs and shaping water conservation strategies that protect reservoir fisheries in the face of climate change. This challenge is explored through a study of striped bass in Lake Texoma. Specifically, a trait‐based approach is utilized to model the amount of water volume associated with suitable habitat conditions for striped bass from 1995 to 2020 through the concept of a temperature–oxygen squeeze. Three main research questions (RQs) will be addressed. First, how is striped bass habitat suitability in Lake Texoma changing seasonally and interannually with a temperature–oxygen squeeze from 1995 to 2020 (RQ1)? Second, what are the combined and individual influences of temperature and DO on suitable habitat volume (RQ2)? Third, what is the impact of a temperature–oxygen squeeze in Lake Texoma on striped bass populations and body condition (RQ3)? Ultimately, this study of striped bass in Lake Texoma intends to provide a framework to explore reservoir fish species dynamics and determine best management practices of reservoir waters under a changing climate.

## 2 Study Area

**FIGURE 1:**
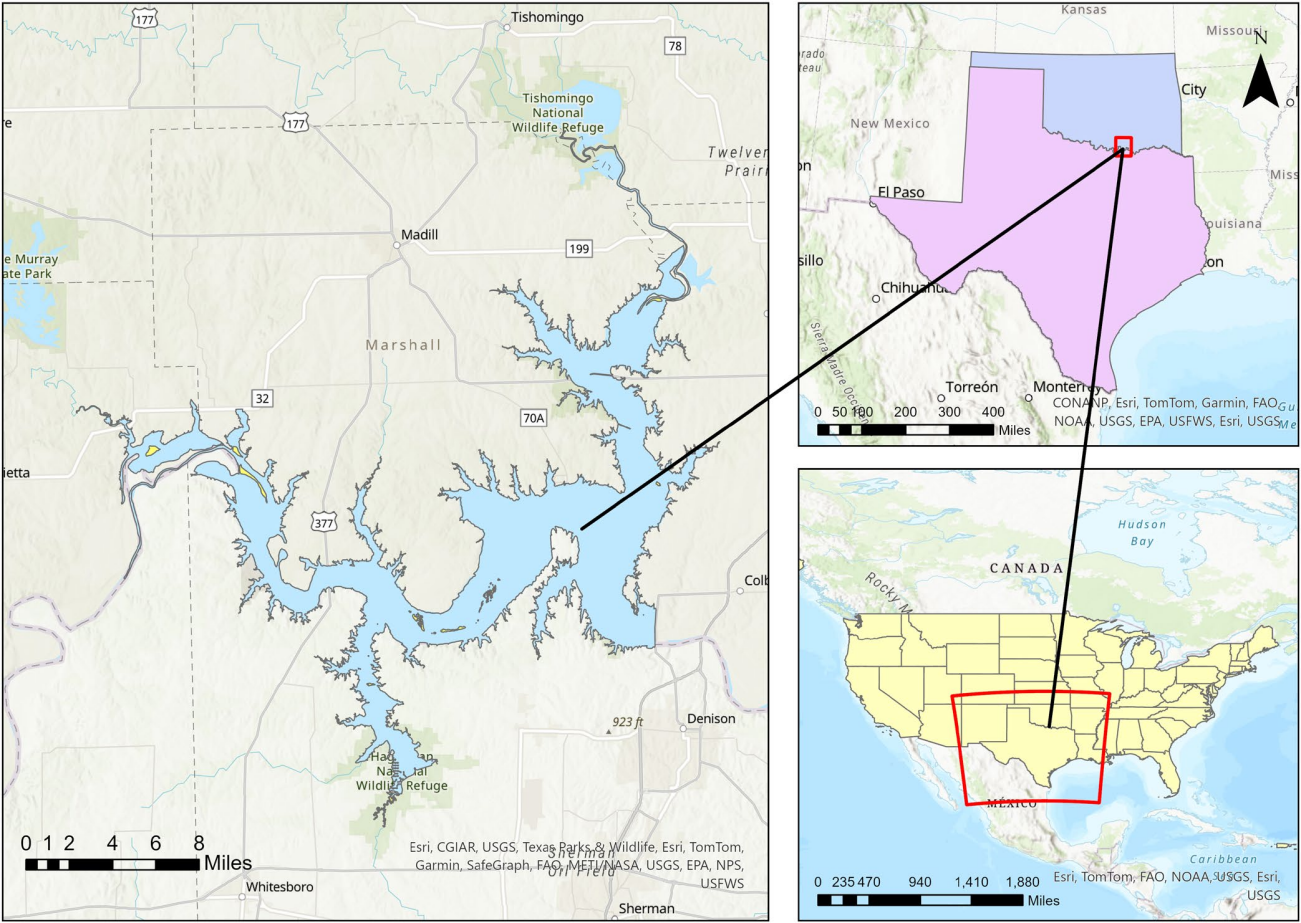
Study area location. Lake Texoma is located on the border of Oklahoma and Texas in the United States and is a major reservoir in the Red River Basin.

The Red River Basin is located in the south‐central United States and flows through New Mexico, Texas, Oklahoma, Arkansas, and Louisiana. It is the second largest river basin by area in the southern Great Plains, draining 169,890 km^2^ (Matthews et al. 2005). The average yearly rainfall ranges from 500 to 1300 mm, with a west‐to‐east gradient, representing multiple ecoregions (U.S. Environmental Protection Agency 2013). Its land cover is dominated by grasslands used for farming, with areas of mixed woodlands and shrublands. The basin has a population of over 4 million people. Situated within the Red River Basin is Lake Texoma, a 36,000 ha impoundment, which lies on the border of Oklahoma and Texas (Figure 1). It was created through the construction of the Denison Dam by the US Army Corps of Engineers (USACE) in 1944 and is the 12th largest reservoir in the United States (Sager et al. 2024). The average water elevation is 188 m above mean sea level (AMSL) with the flood control pool reaching 195 m AMSL. The main basin experiences stratification in the summer from July to mid‐September, as there is oxygen depletion in the hypolimnion (Hubbs 1976). Southwest winds enable dissolved solids in the reservoir waters to be well‐mixed; however, when these winds decrease in the summer, separation and subsequent stratification occur.

## 3 Methods

Our analysis involved three main steps. First, we used modeled profiles of temperature and DO to estimate the daily volume of suitable habitat for striped bass in Lake Texoma for the period of 1995 to 2020. Second, we quantified seasonal and interannual variability in the volume of suitable habitat using a generalized additive model. Third, we fit linear regression models to quantify the relationship between three representations of suitable habitat volume under a summer temperature–oxygen squeeze and striped bass population size and condition during the following winter. These steps were conducted under three potential striped bass thermal limits (25°C, 28°C, and 30°C) and DO conditions of at least 3 mg/L.

### 3.1 Data Collection and Preparation

#### 3.1.1 Deep Learning Model for Temperature and Dissolved Oxygen

**FIGURE 2:**
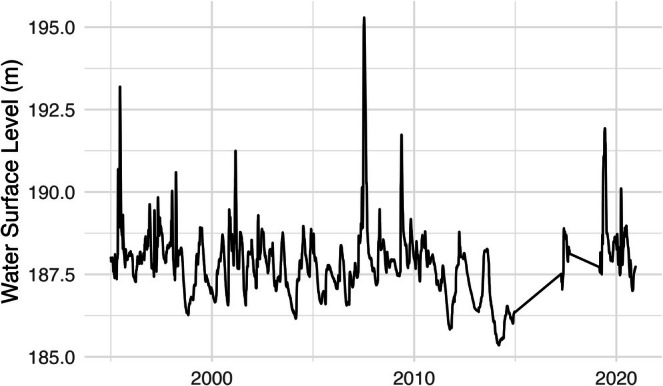
Daily water surface levels for Lake Texoma from 1995 to 2020, collected from Water Data for Texas. Water levels vary by season and by year. Straight lines indicate missing data.

We used a deep learning (DL) model developed by Suaza‐Sierra (2024) that predicted vertical temperature and DO profiles by depth using observed data for training and testing. To develop this model, lake storage data and surface water levels were gathered from the Texas Water Development Board's Water Data for Texas portal and used to validate model outputs (Figure 2). The Texas Water Development Board obtains lake levels from partner organizations that maintain gauges at the reservoirs, taking real‐time water elevation measurements and transmitting them back to those organizations. The USACE site DSNT2 and the United States Geological Survey (USGS) site 07331500 collect measurements for the lake. These calibrated and validated models generated a daily time series of mean water depths and the corresponding temperature and DO concentrations from 0–10 m depths in Lake Texoma.

The Lake Texoma DL model performed well under both stratified and unstratified seasonal conditions (Average Validation Loss = 0.50°C, Root Mean Squared Error = 0.74°C, Mean Absolute Error = 0.50°C, and *R* ^2^ = 0.99). The dates spanned January 1, 1995 to December 14, 2020, with missing values from December 31, 2014 to April 10, 2017 and September 9, 2017 to February 28, 2019. Upon completion of the DL model, Suaza‐Sierra's final dataset included the date, depth (meters), predicted water temperature (°C), predicted DO (mg/L), and surface water level (meters). Every date had temperature and DO values for 0–10 m depths at 1 m intervals from the surface of the lake.

#### 3.1.2 Lake Texoma Bathymetry

To calculate the total volume of suitable striped bass habitat from vertical profiles of temperature and DO, we first created a complete bathymetric raster of Lake Texoma by merging four spatial data sets. A partial bathymetric raster is available from the Texas Water Development Board (2003), but only includes bathymetry to the top of the conservation pool of the reservoir (i.e., up to 188.062 m AMSL). However, water surface elevations during our study period frequently rose above the conservation pool into the flood pool, with a maximum daily surface water level of 195.29 m AMSL. We downloaded three digital elevation model (DEM) tiles from the USGS, National Geospatial Program, and mosaicked them to cover the entire area of Lake Texoma. The bathymetry raster was subtracted from this DEM raster to create a raster that included all possible water elevation levels. The map projection of this raster was UTM Zone 14 N.

### 3.2 Striped Bass Habitat Estimates in Lake Texoma

We calculated the volume of daily suitable habitat available to striped bass using a trait‐based approach, considering specific water temperature and DO requirements. Previous research on the temperature–oxygen squeeze hypothesis suggests that striped bass have a preferred thermal limit of 25°C but can tolerate temperatures as high as 28°C–30°C when avoiding low oxygen conditions of 2–3 mg/L (Coutant 2013). Based on these conclusions, three different thermal limits were considered and DO was consistent across scenarios at a minimum of 3 mg/L to estimate the daily water volume of suitable habitat for striped bass in Lake Texoma: (1) an upper thermal preference range of 25°C and minimum 3 mg/L DO, (2) an upper thermal tolerance range of 28°C and minimum 3 mg/L DO, and (3) an acute upper thermal tolerance range of 30°C and minimum 3 mg/L DO.

Using the modeled vertical profiles of temperature and DO values, and the bathymetric raster, we calculated the surface area at each unique water depth (i.e., a horizontal disc with a height of 1 m that spanned the reservoir) and the suitable water volume within the environmental bounds of striped bass for every day and at each depth interval. Since each cell represents one specific elevation value relative to the water bottom, all areas of the lake in the bathymetry raster at or below each water level were identified. The number of associated raster cells was counted and multiplied by the raster cell area (486.06 × 486.06 m) to determine the water level's surface area. For example, if the water surface level for a given day was 193 m, the model would count all cells in the raster with a value of 193 and below, and multiply the cell count by the cell area to calculate the surface area at a 0 m depth. This process would then be repeated at 1 m intervals from 193 to 183 m, as the hydrologic model had temperature and DO data for the top 10 m of water volume every day. While there is likely more suitable habitat volume beyond 10 m at different times of the year, our models focused on the impact of a summertime temperature–oxygen squeeze. Thus, it was assumed that DO was too low beyond 10 m during the late summer and early fall. At every date and depth, we determined if the given temperature and DO values fell within the defined ranges for each of the three combinations of thermal and oxygen limits. If both values were suitable, the habitat volume at that depth equaled the surface area because depths were at 1 m intervals. If either the temperature or the DO values were outside the defined ranges, the volume was 0. The volumes at 0–10 m depths were then summed to determine the daily habitat volume within the acceptable environmental bounds of striped bass from 1995 to 2020.

### 3.3 Understanding Habitat Changes, Their Drivers, and Impacts on Striped Bass

#### 3.3.1 Temporal Variation in Modeled Suitable Habitat Volume

To address our first research question (RQ1), we analyzed seasonal and interannual changes in the total volume of suitable habitat for striped bass for the years 1995 to 2020. For seasonal changes in habitat suitability, we grouped the data by day of year and calculated the mean and standard deviations of the daily suitable habitat volume for striped bass under the different temperature and DO assumptions.

For long‐term temporal changes in habitat suitability, we used generalized additive models (GAMs) to capture nonlinear seasonal dynamics, long‐term trends, and temporal dependence. First, we checked for autocorrelation and found that the daily habitat volume time series exhibited strong temporal autocorrelation (lag‐1 autocorrelation *ρ*_1_ = 0.97, 0.95, and 0.92 for the upper thermal preference, upper thermal tolerance, and acute upper thermal tolerance ranges, respectively), violating the independence assumption of linear regression models and motivating the use of models that explicitly account for temporal dependence. Thus, we utilized GAMs fitted with the *bam* function in the *mgcv* package in R, which is designed for large time‐series datasets (version 2024.04.2 + 764; R Core Team 2025; Wood 2017). Models assumed a Gaussian error distribution and identity link function and were estimated using fast restricted maximum likelihood (fREML) with discretization enabled for computational efficiency.

For each squeeze range, we fit the following model structure:

$$\begin{array}{c} {\text{Habitat}}_{t}=\beta_{0}+f\left({\text{Habitat}}_{t}-1\right)+f\left({\text{DayOfYear}}_{t}\right)\\ \;+f\left({\text{Date}}_{t}\right)+f\left({\text{WaterLevel}}_{t}\right)+\varepsilon_{t} \end{array}$$

where $f\left({\text{Habitat}}_{t}-1\right)$ is a smooth term of the lagged response variable (one‐day lag) to account for temporal autocorrelation in daily habitat volume, $f\left(\text{DayOfYear}\right)$ is a cyclic cubic regression spline capturing within‐year seasonal structure, $f\left(\text{Date}\right)$ is a smooth term capturing long‐term trends in habitat availability through time (with time expressed as fractional years, i.e., 1995.003 is January 1, 1995, and so on), $f\left(\text{WaterLevel}\right)$ represents the effect of daily water surface elevation (m AMSL) on habitat volume, and $\varepsilon_{t}$ is the residual error term. Smooth terms were fit using thin‐plate regression splines, except for day of year, which was modeled using a cyclic cubic spline to enforce continuity between the end and beginning of the calendar year. Basis dimensions (k) were set conservatively, and effective degrees of freedom (edf) were used to assess the complexity of fitted relationships. Partial effect plots were examined to interpret the independent contribution of each predictor after accounting for other model components.

#### 3.3.2 Influence of Environmental Variables on Habitat Suitability

To address our second research question (RQ2), we manipulated the habitat model to determine which environmental stressor (temperature or DO_3mg/L_) was a greater driver of striped bass habitat suitability under three thermal limits for the temperature–oxygen squeeze ranges. Similar to the daily suitable habitat volume calculation under each temperature–oxygen squeeze range, we quantified daily habitat volume when neither stressor was considered, only DO was considered, only temperature was considered, and both stressors were considered. The mean daily suitable habitat volume was calculated for each of these scenarios and plotted. This allowed for the visual comparison of suitable water volume under each stressor scenario and squeeze range to better understand how the environmental stressors influenced habitat suitability for striped bass in Lake Texoma. The environmental stressor with the greatest difference between total available habitat volume in the first 10 m and suitable habitat volume was considered to have more influence on habitat suitability within that squeeze range.

#### 3.3.3 Impacts of a Temperature–Oxygen Squeeze on Striped Bass

To address our third research question (RQ3), we fit linear regression models to determine if there was a relationship between the modeled volume of suitable habitat under a temperature–oxygen squeeze and striped bass population sizes and body condition in the following winter. The daily suitable habitat volume estimates were further used to calculate three habitat indicators for each year that were representative of the modeled temperature–oxygen squeeze. The first value was the yearly minimum habitat volume. This represents an abrupt temperature–oxygen squeeze or “pulse” disturbance for the striped bass (Bender et al. 1984). The second value was the minimum average volume across a 14‐day rolling period. Starting at the beginning of the year, the volumes associated with the first 14 days were averaged. Moving to the second day of the year, the volumes of the following 14 days were averaged, and so on throughout the year. Of those 14‐day averages, the minimum value was selected for each year. Similarly, the third value was the minimum average volume across a 30‐day rolling period. These two values represent variations of a long‐term temperature–oxygen squeeze or “press” disturbance for striped bass (Bender et al. 1984). The maximum press disturbance was set to 30 days because some studies have shown that striped bass can tolerate water temperatures above their thermal preference, from 25°C to 29°C, for periods of up to one month (Coutant 2013; Zale et al. 1990). We calculated these three indicators of habitat volume for each of the striped bass temperature and DO squeeze ranges, resulting in nine independent variables for the linear regression models.

**FIGURE 3:**
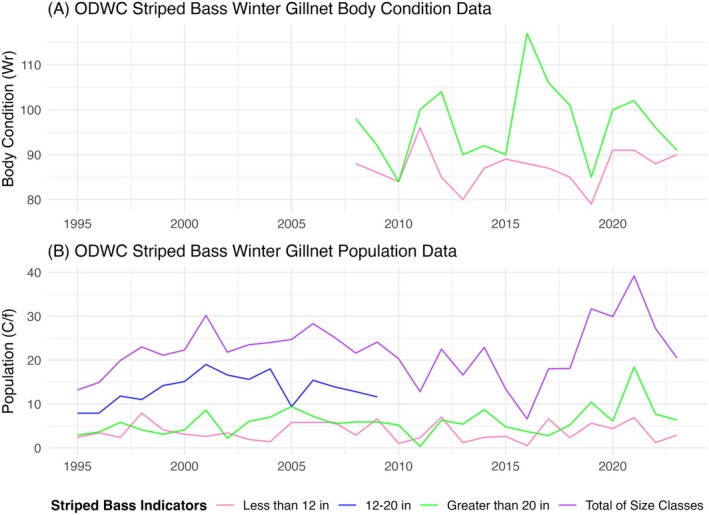
Yearly striped bass winter gillnet data collected by the ODWC. The ODWC recorded (A) relative weights (Wr) and (B) fish catch per net night (C/f) for three size classes of < 12 in, 12–20 in, and > 20 in. Wr represents striped bass body condition and C/f represents striped bass population.

The dependent variables of population and body condition were gathered from the Oklahoma Department of Wildlife Conservation (ODWC) Lake Texoma Fisheries Management Plan (Sager et al. 2024; Figure 3). The plan contains data for the total number, fish catch per net night (C/f), and relative weights (Wr) of < 12 in, 12–20 in, and > 20 in size groups for striped bass from 1982 to 2023. Size classes (< 12 in, 12–20 in, > 20 in) and fisheries metrics (C/f and Wr) follow ODWC and Texas Parks and Wildlife Department (TPWD) monitoring and management practices and were used here to maintain consistency with the original dataset. Differences in data collection and reporting between years likely reflect staffing changes and updates to data management tools (M. Mauck, ODWC, Personal Communication, 2026). Relative weights are a measure of fish wellbeing and were quantified by the ODWC according to methods outlined by Brown and Murphy (1991) for striped bass (Smith 2012). For the fish catch per net night, data were available for all years and all size groups, except for 12–20 in groups after 2009. Additionally, data were only available for relative weights from 2008 to 2023 for < 12 in and > 20 in size groups. Since 1993, this data has been jointly collected by the ODWC and TPWD through winter gillnetting samples for a couple weeks in February. This resulted in six dependent variables used in the models: (1) the yearly total catch per net night, (2) the catch per net night of fish < 12 in, (3) the catch per net night of fish 12–20 in, (4) the catch per net night of fish > 20 in, (5) the relative weights of fish < 12 in, and (6) the relative weights of fish > 20 in. Thus, with the 9 independent variables and 6 dependent variables, we ran 54 total linear regression models to evaluate the impacts of a temperature–oxygen squeeze on striped bass population and body condition, with the yearly striped bass descriptor as a function of the temperature–oxygen squeeze habitat volume from the previous summer (Table 1). To reduce Type 1 errors (i.e., incorrectly rejecting the null hypothesis) across 54 linear regression models, we used the Bonferroni correction, where *α* is divided by the number of tests (Weisstein 2004). After applying this correction, our original *α* of 0.05 was reduced to a new *α* of 0.00093, and we used this smaller value to determine model significance.

**TABLE 1:** Different combinations of temperature–oxygen squeeze ranges, indicators of summer habitat volume resulting from a temperature–oxygen squeeze, and striped bass descriptors of population and body condition used to conduct 54 linear regression models. All regressions were run at an annual scale.

Temperature–oxygen squeeze range	Habitat indicators	Striped bass descriptors
Upper thermal preference (25°C and 3 mg/L)	Minimum habitat volume (m^3^)	Total Fish per Net Night (C/f)
		Fish per Net Night (C/f) of < 12‐in. Striped Bass
Upper thermal tolerance (28°C and 3 mg/L)	Minimum average habitat volume of a 14‐day period (m^3^)	Fish per Net Night (C/f) of 12–20‐in. Striped Bass
		Fish per Net Night (C/f) of > 20‐in. Striped Bass
Acute upper thermal tolerance (30°C and 3 mg/L)	Minimum average habitat volume of a 30‐day period (m^3^)	Relative Weights (Wr) of < 12‐in. Striped Bass
		Relative Weights (Wr) of > 20‐in. Striped Bass

## 4 Results

### 4.1 Seasonal Habitat Suitability

**FIGURE 4:**
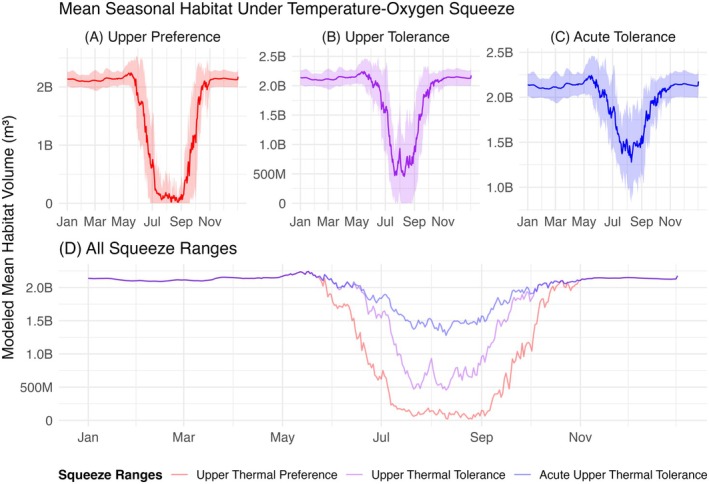
Mean seasonal habitat volume suitable for striped bass in Lake Texoma based on three temperature–oxygen squeeze ranges: (A) upper thermal preference (25°C and 3 mg/L), (B) upper thermal tolerance (28°C and 3 mg/L), and (C) acute upper thermal tolerance (30°C and 3 mg/L). (D) Each squeeze range resulted in different volumes of suitable habitat in the summer. In all panels, solid lines display the mean value calculated across years 1995–2020. In panels (A–C), the shaded envelopes display standard deviation across those years.

We found consistent seasonal trends in the volume of preferred habitat for striped bass in Lake Texoma (Figure 4; RQ1). Based on the three defined temperature and DO ranges, the modeled mean suitable habitat volume remained relatively stable in the winter and spring months, declined in early summer, reached lows in late summer and early fall, and rose again in late fall (Figure 4). On average, the model showed that Lake Texoma had a maximum volume of over 2 billion m^3^ in the first 10 m of water depth from the surface. Under the acute upper thermal tolerance range, the average water volume suitable for striped bass decreased by about one quarter to less than 1.5 billion m^3^ in the summer (Figure 4C). For the upper thermal tolerance range, average suitable summer habitat volume decreased by about three quarters to 500 million m^3^ (Figure 4B). For the upper thermal preference range, average suitable summer habitat volume decreased to nearly 0 m^3^, meaning that there was little to no suitable habitat in the reservoir (Figure 4A). Overall, the results of our model demonstrated that a temperature–oxygen squeeze occurs in Lake Texoma each summer, and more strict biological requirements leave striped bass with less suitable water volume.

### 4.2 Temporal Dynamics of Habitat Suitability

**FIGURE 5:**
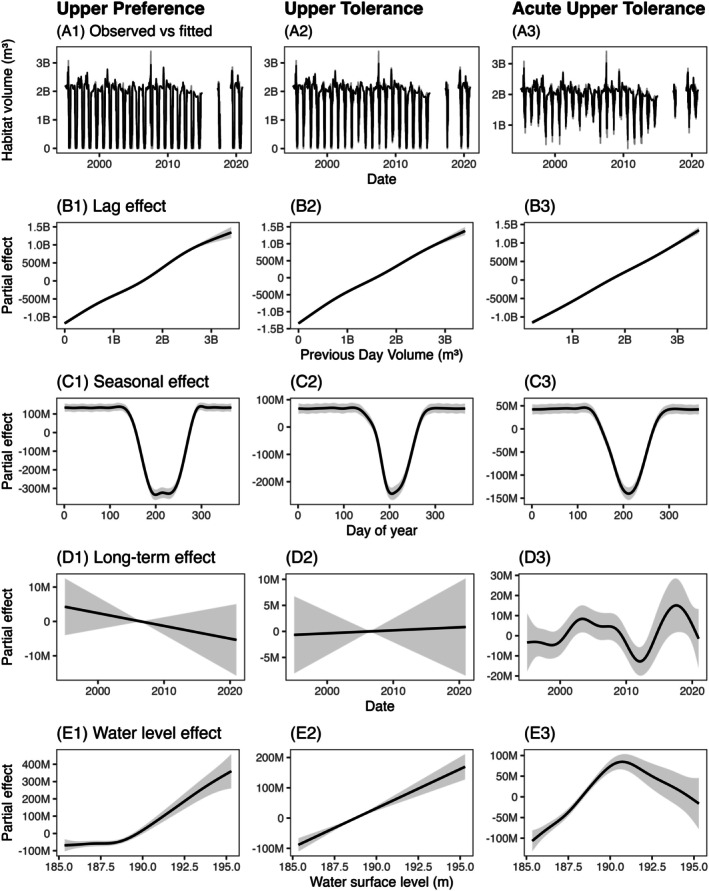
Generalized additive models (GAMs) depicting overall model fit (A1–3) and smooth functions of the partial effect of covariates on daily suitable habitat volume. Covariates include day‐to‐day dependence (B1–3), seasonality (C1–3), long‐term trends (D1–3), and water level (E1–3) for each temperature–oxygen squeeze range of upper thermal preference (column 1), upper thermal tolerance (column 2), and acute upper thermal tolerance (column 3). In the full GAM, gray lines represent observed data and black lines represent the fitted model. The plots for each covariate show 95% confidence intervals in gray and the estimated smooth effect in black.

GAMs explained a substantial proportion of the variability in daily suitable habitat volumes for the upper thermal preference, upper thermal tolerance, and acute upper thermal tolerance ranges (*R* ^2^ = 0.94, 0.91, and 0.86, respectively; Figure 5A). The one‐day lagged habitat volume was a highly significant predictor of habitat volume in each model, indicating strong temporal persistence (*p* < 0.001; *F* = 3136.54, 4159.53, and 3203.80). While the relationship between habitat volume and lagged habitat was nonlinear (edf = 3.88, 3.85, and 3.57), the plotted smooth trend showed close to proportional relationships between one day's suitable habitat volume and the previous day's suitable habitat volume (Figure 5B). Nonlinear trends can be explained by the influence of seasonal stratification dynamics on habitat suitability.

Seasonality was also a significant predictor of habitat volume across all thermal thresholds (*p* < 0.001). Cyclic smooths showed a prominent nonlinear mid‐summer contraction in suitable habitat volume (edf = 14.74, 14.06, and 11.74; *F* = 47.60, 34.08, and 33.79; Figure 5C). Furthermore, the magnitude of the squeeze differed between each range, with the upper thermal preference experiencing many 0 volume days, the upper thermal tolerance experiencing fewer 0 volume days, and the acute upper thermal tolerance experiencing no 0 volume days.

Long‐term interannual smooths differed between squeeze ranges. The upper thermal preference range showed an insignificant negative linear trend, the upper thermal tolerance range showed an insignificant near flat linear trend, and the acute upper thermal tolerance range showed a significant nonlinear trend (*p* = 0.30, 0.86, and 0.005; edf = 1.00, 1.00, and 6.56; Figure 5D). Insignificant linear trends were likely driven by the more restrictive thermal thresholds, which often caused the complete depletion of daily suitable habitat volume. The significant nonlinear pattern indicated dips and recoveries over time, with a large decline in suitable habitat volume around 2010 and increased volume leading up to 2020.

Finally, the partial effect of water level also differed between squeeze ranges. The upper thermal preference range showed a significant nonlinear trend, the upper thermal tolerance range showed a significant linear trend, and the acute upper thermal tolerance range showed a significant nonlinear trend (*p* < 0.001; edf = 4.33, 1.00, and 5.43; Figure 5E). The upper thermal preference and upper thermal tolerance both showed that as water level increased, so did suitable habitat volume, with the preference range having more of an exponential trend, and the tolerance range having a more proportional relationship between water level and habitat volume. The acute upper thermal tolerance range indicated a different relationship where, as water level increased, so did suitable habitat volume, until water level reached approximately 191 m and suitable habitat volume began to decrease as water level increased. These differences suggest that hydrologic expansion of the upper 10 m disproportionately affects suitable habitat volume defined by the most and least restrictive thermal limits, while the intermediate threshold responds more uniformly.

### 4.3 Influence of Temperature and DO on Habitat

**FIGURE 6:**
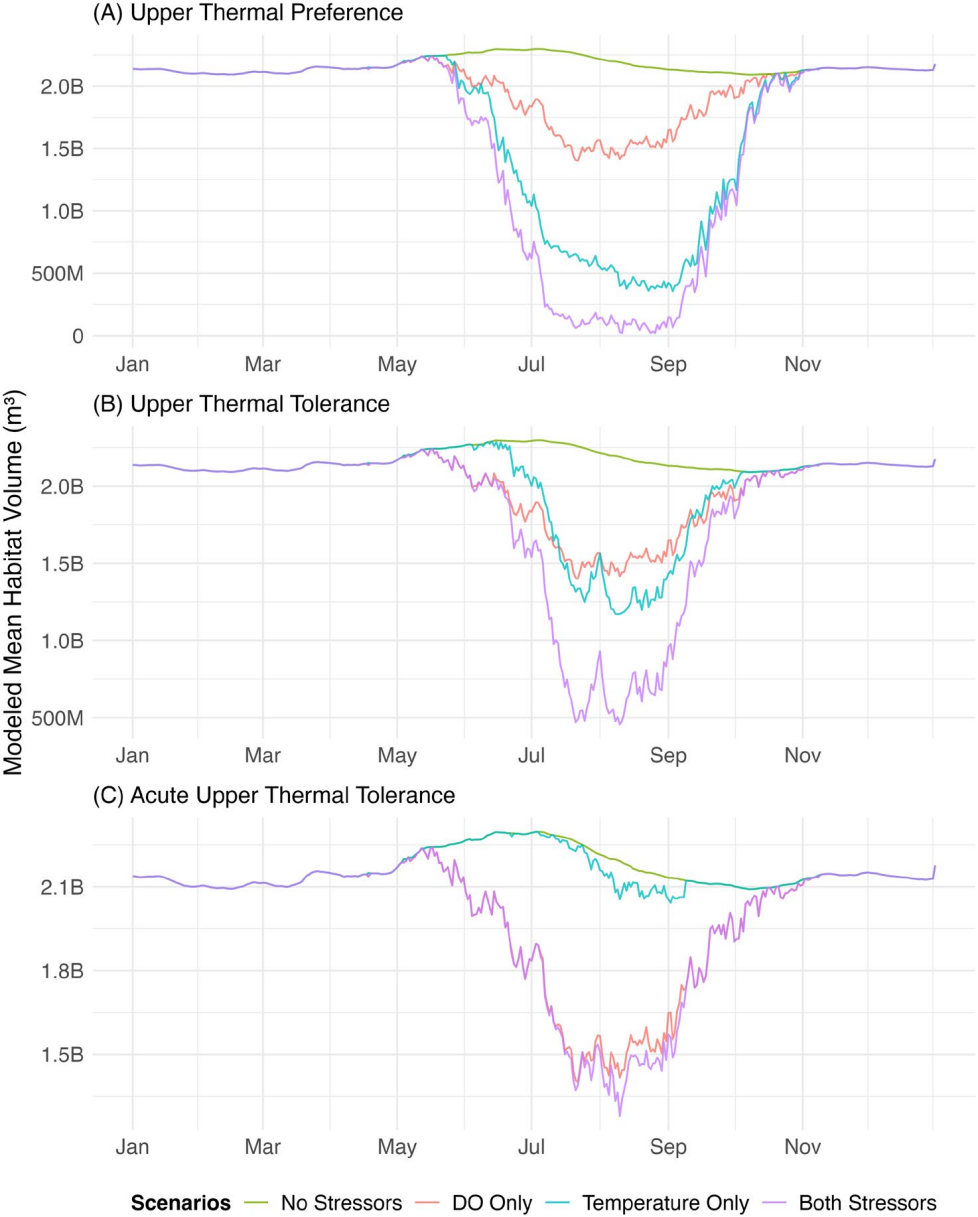
Influence of environmental drivers (temperature and DO) on mean seasonal habitat volume suitable for striped bass in Lake Texoma based on the (A) upper thermal preference range, (B) upper thermal tolerance range, and (C) acute upper thermal tolerance range. Each panel shows the total volume of water in the top 10 m of the reservoir (the “No Stressors” scenario in green) and the suitable habitat volumes calculated by considering only DO limits (“DO Only” in orange), only temperature limits (“Temperature Only” in blue), and both DO and temperature (“Both Stressors” in purple).

Fluctuations in available habitat were driven more often by meeting the DO threshold than by the thermal thresholds (Figure 6; RQ2). Under the acute upper thermal tolerance range, DO was the dominant driver of suitable habitat volume, with temperature exhibiting little influence, as the amount of habitat volume available when constrained by DO alone was almost the same as the amount of habitat volume available when constrained by both DO and temperature combined (Figure 6C). For the upper thermal tolerance range, temperature and DO had a similar influence on suitable habitat volume as they both resulted in around one quarter to two fifths less suitable volume in the summer compared to the control (Figure 6B). In early summer, DO was a slightly greater driver, and in late summer and early fall, temperature was a slightly greater driver. However, for the upper thermal preference range, temperature was a greater driver than DO as it resulted in about three quarters less water volume of suitable habitat in the summer compared to the control, whereas the DO level threshold only reduced suitable habitat volume by about one quarter (Figure 6A). In this range, the differences in suitable habitat between temperature‐only and DO‐only scenarios were not as severe as the differences in the acute upper thermal tolerance range, which showed the thermal threshold exhibiting very little to no influence on habitat. Generally, temperature and DO influenced suitable habitat volume differently depending on the temperature–oxygen squeeze and thermal limits of striped bass.

### 4.4 Impacts of a Temperature–Oxygen Squeeze on Striped Bass

**FIGURE 7:**
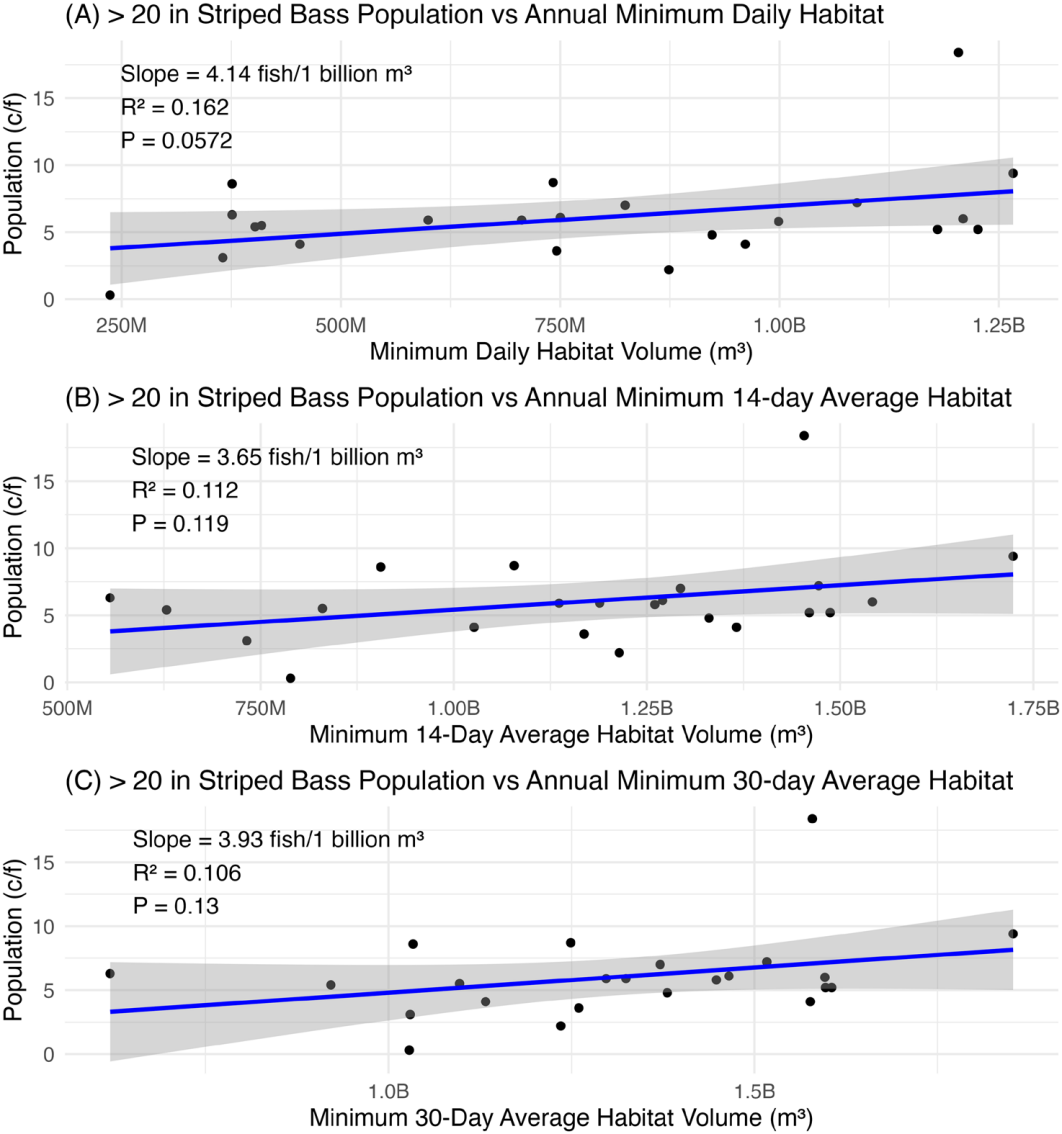
Three examples of linear regression models investigating the relationships between the yearly catch (C/f) of greater than 20 in striped bass and (A) the yearly minimum daily habitat volume, (B) the yearly minimum average volume of a 14‐day rolling period, and (C) the yearly minimum average volume of a 30‐day rolling period under the acute upper thermal tolerance range.

Despite dramatic reductions in the water volume of habitat suitable for striped bass each summer (Figures 4 and 5), we found no evidence for a relationship between the yearly ODWC striped bass winter gillnet data (i.e., fish catch per net night and relative weights of different size classes) and yearly indicators of modeled suitable habitat volume under a temperature–oxygen squeeze (RQ3). The 54 linear regression models showed *p*‐values ranging from 0.047 to 0.978, meaning that, at a Bonferroni‐corrected alpha of 0.00093, no models were statistically significant. Figure 7 shows examples of the modeled trends for C/f of > 20 in striped bass and each of the three representations of a temperature–oxygen squeeze under the acute upper thermal tolerance range.

## 5 Discussion

This study provides valuable insights into modeling freshwater habitat suitability for striped bass in Lake Texoma under a temperature–oxygen squeeze, while also exploring whether such a squeeze affected striped bass populations and body condition in Lake Texoma from 1995 to 2020. The modeled habitat available to striped bass in Lake Texoma changed seasonally, with less suitable habitat volume available in the summer within striped bass' environmental bounds. Partial effects of seasonality, day‐to‐day persistence, and reservoir water level significantly influenced model results. However, insignificant interannual linear trends and a significant nonlinear trend were found for the effect of time, indicating that suitable summer habitat volume due to stratification has not experienced a significant proportional change across years. This model also revealed differences in the strength of environmental drivers, with temperature being a stronger driver of habitat suitability than DO in the upper thermal preference range, temperature and DO being of similar influence in the upper thermal tolerance range, and DO being a stronger driver than temperature in the acute upper thermal tolerance range. Finally, despite annual seasonal changes in the modeled volume of suitable habitat, annual striped bass populations were not significantly impacted by the summer temperature–oxygen squeeze, suggesting that a complex set of processes may be influencing striped bass dynamics in Lake Texoma.

Overall, our models demonstrated that over more than two decades, modeled habitat volume, based on DO and temperature conditions suitable for striped bass, experienced regular seasonal changes. Specifically, the model showed that more restrictive thermal thresholds caused a floor effect where summertime suitable habitat was frequently limited or nonexistent, so changes in water level that increase basin area and volume can have a big effect on suitable habitat volume when thermal and DO criteria are met (Quillfeldt 2022). However, at a higher thermal threshold, habitat suitability dynamics are structure‐dominated. At higher water levels, stratification intensifies, sharpening thermal gradients, reducing mixing of layers, and accelerating oxygen depletion, which results in less suitable water volume (Birt et al. 2025; Jane et al. 2023; Kraemer et al. 2015). These dynamics may be exacerbated by climate change in the future, as warmer surface water temperatures hold less DO than cooler temperatures (Jane et al. 2021). A study of 57 Texas reservoirs, including Lake Texoma, determined that temperature and DO were correlated to atmospheric predictors, and global climate change is expected to increase water temperatures and decrease levels of DO in Texas reservoirs (Gelca et al. 2016). Thus, climate‐related changes in water level, air temperature, thermocline, and chemocline in reservoirs have the potential to alter water quality and habitat structure for striped bass and other freshwater fishes. Such alterations can reduce survival of certain fish species and facilitate changes in fish assemblages, geographic distributions, and invasions by nonnative fishes (Budnik et al. 2021; Lynch et al. 2016; Miranda et al. 2020). An uncertain climate and its potential to change ecological systems is especially important to consider in water management decisions of already water‐limited regions like the Red River Basin.

While the hydrologic and habitat suitability models demonstrate that a temperature–oxygen squeeze is annually occurring in Lake Texoma during the summer, our study showed no indication that a temperature–oxygen squeeze has significantly influenced long‐term striped bass population and body condition patterns. In accordance with this finding, Matthews et al. (1985) observed that striped bass exposed to high water temperatures did not have any apparent mortalities and were robust with well‐developed reproductive organs around the time of their study from 1982 to 1984. Other studies in Oklahoma and Texas reservoirs demonstrated that striped bass could survive high temperatures for about one month with a sufficient bioenergetic balance (Farquhar and Gutreuter 1989; Zale et al. 1990). Like Texoma, systems without true refuges have generally shown stable striped bass populations during the summer temperature–oxygen squeeze (Coutant 2013). Furthermore, the ODWC and TPWD have continuously made changes to length and bag limit restrictions on striped bass in Lake Texoma to meet the needs of both the fishery and angling public, resulting in a relatively stable striped bass population since the establishment of current regulations in 1996 (Sager et al. 2024). Conversely, many studies of different reservoirs in the southern United States have shown evidence of summer mortality, disease, and malnutrition of large adult striped bass due to the squeeze (Coutant 1978, 2013; Lewis 1985; Lewis et al. 1979; Matthews 1985; Young and Isely 2004). Therefore, studies in southern United States reservoirs have differing conclusions regarding the impacts of a temperature–oxygen squeeze on striped bass, possibly due to differences in fisheries management efforts and striped bass adaptability.

One hypothesis for why we did not find evidence of temperature–oxygen squeeze impacts is that, without a significant long‐term linear decline in habitat suitability, striped bass might have become adapted to the annual stratified summer conditions in Lake Texoma, enabling them to tolerate warm water temperatures and low DO concentrations. The adaptability of striped bass in Lake Texoma specifically could be driven by the lake morphology, which lacks refuge sites with cool, oxygenated waters (Coutant 2013; Matthews et al. 1989; Summers 1982). In these systems without true refuges, striped bass have successfully inhabited mid‐depths at temperatures above their preference with no apparent mortalities but sometimes resulted in poor body condition (Farquhar and Gutreuter 1989; Grimes 1987, 1993; Jackson and Hightower 2001; Matthews et al. 1985, 1989). Additionally, conversations with the ODWC region fisheries supervisor, Matt Mauck, revealed that the lake destratifies in late September or early October, around the time that striped bass become overly stressed, allowing them to narrowly avoid detrimental impacts and further implying that they can tolerate high temperatures for a limited period in the summer (M. Mauck, ODWC, Personal Communication, 2025). Another possible explanation is the timing of striped bass monitoring. While the ODWC and TPWD conduct yearly gillnet sampling, it is done in the winter instead of the summer or fall when lake stratification occurs, causing the temperature–oxygen squeeze (Hubbs 1976; Sager et al. 2024). Sampling of adult striped bass in Lake Buchanan, Texas, found that relative weights increased significantly per day from summer to early spring (Smith 2012). Consequently, the effects of a temperature–oxygen squeeze on striped bass may not have been fully captured in the models, as body conditions could have improved by the time late winter sampling was conducted.

Another hypothesis is that factors other than a temperature–oxygen squeeze could be influencing striped bass populations and body conditions to such an extent that they mask the effect of the squeeze. The ODWC has identified a combination of potential drivers of striped bass populations, such as the discharge of flood waters, winter kills of threadfin shad (a primary forage species), algal blooms, overcrowding, drought, limited spawning opportunities, angler harvest, and catch and release mortality (Sager et al. 2024). Winter kills of threadfin shad can cause abrupt, system‐wide collapses of the forage base before the growing season, strongly limiting striped bass growth and year‐class strength in the following year (M. Mauck, ODWC, personal communication 2025), whereas summer mortality is more episodic and confounded with direct thermal and oxygen stress on striped bass (Adams et al. 1982; Sager et al. 2024). Studies by the ODWC reported significant striped bass die‐offs and a decrease in fish catch in Lake Texoma both in 2011 after a harsh winter killed threadfin shad and from 2014 to 2016 following a severe multi‐year drought (Figure 3; Sager et al. 2024). Some of these issues will be further exacerbated by climate change, underscoring a need to recognize the most influential combination of factors on striped bass dynamics.

These factors can also be related to the effects of a temperature–oxygen squeeze, demonstrating the complexity of processes shaping reservoir fish populations. Previous studies of the effects of a temperature–oxygen squeeze on striped bass have revealed that survival is related to the duration of exposure and bioenergetic balance, meaning that greater food availability can help mitigate the impacts of long‐term exposure to a squeeze (Coutant 2013; Thompson and Rice 2013; Zale et al. 1990). This provides another possible explanation for why we did not find a significant relationship between the temperature–oxygen squeeze and striped bass population in Lake Texoma. However, increased drought and heat waves in the future under climate change can intensify annual water level fluctuations and expose fish to higher water temperatures, which could exacerbate the squeeze and greatly influence striped bass survival (Barrett et al. 2024; Miranda et al. 2020). Air temperatures have become warmer across the Red River Basin and are projected to increase up to 6°C–7°C by the end of the century in multiple global climate models and high emissions scenarios, increasing water surface temperatures (Bertrand and McPherson 2019). Future models of habitat suitability should account for the interaction of multiple factors on striped bass population dynamics, informing best management practices for fisheries and water resources in reservoirs under climate uncertainty.

Limitations of this study include potentially insufficient data and model assumptions like linearity and outliers. The hydrological model was missing some temperature and DO data in the years of 2015 to 2019. Additionally, sampling of striped bass size groups and analysis by the ODWC and TPWD has changed interannually, resulting in only 11–23 observations per indicator of striped bass population and body condition (Sager et al. 2024). The missing data could have influenced the results of this study by altering the estimates of suitable habitat volume under each temperature–oxygen squeeze range and impacting model results and insights. Furthermore, the striped bass models assume that the relationships are linear and do not consider outliers in winter gillnet samples or in changes to habitat volume associated with extremes in water level from flood or drought events (Figures 2 and 3). Despite these limitations and insignificant relationships, it is understood that the temperature–oxygen squeeze hypothesis has mixed support (Coutant 2013). Conclusions from previous studies conducted in Lake Texoma and insights from Lake Texoma fisheries managers support the idea that a temperature–oxygen squeeze is occurring in the lake but might not be significantly influencing striped bass populations and body condition.

Understanding how future climate change will impact striped bass habitat in reservoirs is imperative for managers of reservoir fisheries. Based on striped bass tolerances to extreme water quality conditions, water management strategies can be developed to control DO and temperature in Lake Texoma to some extent. Reducing nutrient runoff into the reservoir and maintaining water levels can help prevent an increase in algal blooms, which further decrease DO concentrations (Yasarer and Sturm 2016). Optimization models can be created to manage water withdrawals in the reservoir that control the thermal and chemical regimes for fisheries needs while balancing human water needs (He et al. 2022; Weber et al. 2017). Additionally, managers like Mauck believe that temperature is limiting their ability to grow large fish in Lake Texoma. While conditions that allow striped bass to reach their historic trophy sizes have been lacking, recreational fishing regulations and improved harvest of small adult fish have been implemented to maintain stable populations of the species and can be further improved in the future (Coutant 2013; Sager et al. 2024). These monitoring and managing tools can reduce anthropogenic stressors, restore key reservoir features, and provide more refugia habitat for striped bass (Miranda et al. 2020).

Our habitat suitability model represents a novel approach that uses species‐specific traits and daily models of reservoir conditions to identify limiting habitats for freshwater fishes and allows us to better understand how they might be impacted by climate change and intensifying stratification. By recognizing that less suitable water conditions are expected in the future in large reservoirs located in the humid subtropical region of the United States, reservoir fisheries managers can better prepare for the management and conservation of economically and ecologically important fish species (Gelca et al. 2016; Miranda et al. 2020). Water is typically managed as a physical resource for human use, often overlooking its vital interconnections across ecosystems and its essential importance to other living organisms. Hence, a more holistic water management strategy is needed to protect freshwater habitats (van Rees 2024). Lessons learned from local and regional scale studies can provide useful insights into designing such management strategies. Our study of Lake Texoma's fishery serves as an important study site as it has a rare, self‐sustaining striped bass population that attracts millions of visitors annually and contributes substantially to local economies. However, increasing regional water demand has raised concerns about maintaining adequate water quality and the potential negative impacts on aquatic organisms and recreational activities (Sager et al. 2024). To address these challenges, reservoir and fisheries managers must work together to assess the trade‐offs in allocating water for human uses and fisheries needs, particularly in water‐limited regions. Thus, the long‐term sustainable management of freshwater resources and biodiversity is essential both locally and globally.

## 6 Conclusion

Reservoir fisheries provide irreplaceable benefits to communities and the environment, posing a need to understand how they may be affected by changes in water quality. In Lake Texoma, striped bass, a species of economic, recreational, and ecological importance, are exposed to a temperature–oxygen squeeze, which is expected to exacerbate with rising water temperatures and decreased DO concentrations in the future. The use of a reservoir water quality model and habitat suitability model allowed for the identification of potentially limiting habitats under various temperature–oxygen squeeze scenarios. While the modeled suitable habitat volumes were not significantly related to striped bass populations and body condition in Lake Texoma, mortalities have been observed and striped bass are no longer reaching historical trophy sizes in the reservoir. Thus, further work should be conducted to determine primary factors influencing striped bass populations and sizes in Lake Texoma and aid the ODWC and TPWD in their efforts to maintain a successful striped bass fishery under an uncertain climate. Future work could include targeted summer sampling of striped bass in Lake Texoma, expanding the model to account for other environmental factors and their combined effect on striped bass, and using this study as a framework to investigate the impacts of temperature–oxygen squeeze on other reservoirs and fish species. Continued research can provide a better understanding of environmental stressors and guide sustainable fisheries management and conservation in major southern and south‐central United States reservoirs.

## Author Contributions

**Ally M. Whiteis:** conceptualization (equal), data curation (equal), formal analysis (equal), methodology (equal), validation (equal), visualization (equal), writing – original draft (lead), writing – review and editing (equal). **Shadi Fathollahifard:** data curation (equal), formal analysis (supporting), methodology (supporting), validation (supporting), visualization (supporting), writing – review and editing (supporting). **Isabela Suaza‐Sierra:** data curation (equal), methodology (supporting), validation (supporting), writing – review and editing (supporting). **Hernan A. Moreno:** conceptualization (equal), funding acquisition (equal), methodology (equal), supervision (equal), validation (equal), writing – review and editing (equal). **Nishan Bhattarai:** formal analysis (supporting), supervision (equal), validation (equal), writing – review and editing (equal). **Thomas M. Neeson:** conceptualization (equal), formal analysis (equal), funding acquisition (equal), methodology (equal), supervision (lead), validation (equal), visualization (equal), writing – review and editing (equal).

## Funding

This work was supported by University of Oklahoma Libraries and South Central Climate Adaptation Science Center (20006684).

## Conflicts of Interest

The authors declare no conflicts of interest.

## Data Availability

Data used for this study are available for download from Dryad: https://doi.org/10.5061/dryad.3r2280gwb.
